# Genetic Diversity and Structure Analysis of Wild Oysters (*Crassostrea virginica*) Within and Between River Systems Reveals Spatial Genetic Structure and Salinity‐Adaptive Variation Across the Canadian Maritimes

**DOI:** 10.1111/eva.70338

**Published:** 2026-10-22

**Authors:** Stephanie Hall, Denise Méthé, Royce Steeves, Mark LaFlamme, Curtis Dinn, Rémi Sonier, Jeff C. Clements

**Affiliations:** ^1^ Fisheries and Oceans Canada, Gulf Region Moncton New Brunswick Canada

**Keywords:** aquaculture, eastern oyster, population genetics, restoration, shellfish

## Abstract

In the Canadian Maritimes, the eastern oyster ( *Crassostrea virginica* ) industry has been shaped by population‐scale events, including ~90% reductions of natural beds from Malpeque disease. For many years, Maritime oyster populations were considered genetically homogenous due to large‐scale transplants to rehabilitate depleted beds; however, recent studies suggest that ancestral populations survived. We characterized genetic diversity and geographical genetic structure (using ddRAD‐seq) of wild oysters from 24 locations (*n =* 1409) across New Brunswick (NB), Prince Edward Island (PEI), and Nova Scotia (NS), including multiple locations along estuarine gradients within the same river systems. While genetic differentiation was low  between Maritime locations (*F*_st_ = 0.012), ADMIXTURE analysis demonstrated spatial structure with cross‐validation (CV) error optimized at *K = 4* ancestral populations. Oysters from the Bras d'Or Lakes in NS, and Miramichi and Richibucto in NB were characterized by distinct dominant ancestries, whereas populations from Bouctouche and Caraquet in NB, the Foxley, Grand, and Brudenell Rivers on PEI, and Pictou in NS shared a common dominant ancestry. Populations from coastal Cape Breton (Aspy River and Arichat) in NS exhibited mixed ancestry, primarily consisting of Bras d'Or Lake and ‘Gulf’ lineages. Within estuaries, genetic differentiation between upriver and downriver sites was minimal (*F*_st_ = 0.002–0.003), suggesting ongoing gene flow along estuarine gradients; however, each estuary contained a subset of SNP loci putatively under selection, indicating potential local adaptation despite high connectivity. Functional annotation of candidate loci identified genes associated with membrane transport, cellular stress response, protein turnover, and metabolic pathways, suggesting that environmental gradients may contribute to adaptive divergence. Ultimately, this reveals a complex genetic landscape and demonstrates that oyster beds in the Maritime provinces have retained genetic structure from both historical transfer activity in the region and local adaptation. The results provide important insights for the conservation, management, and restoration of eastern oyster populations throughout Atlantic Canada.

## 1 Introduction

The Canadian Maritime provinces have a long history of transferring eastern oysters ( *Crassostrea virginica* ) between river systems on the eastern seaboard. Historically, transfers were carried out primarily to compensate for habitat degradation, overfishing, and disease pressures (Ingersoll 1881; Logie 1958; Medcof 1961; Drinnan and England 1965; Drinnan 1967; MacKenzie 1996). Examples of such transfers include large‐scale restoration efforts carried out after oyster populations succumbed to Malpeque disease in the 1930s–1960s (Needler and Logie 1947; Logie 1958; Drinnan and England 1965). Malpeque disease, caused by an unknown aetiologic agent, decimated natural beds with ~90% cumulative mortality (Bower and McGladdery 1994) and not only affected the coastal and estuarine ecosystems, but resulted in significant socioeconomic loss (Bower and McGladdery 1994). Prior to the appearance of Malpeque disease, annual landings from commercial harvest of natural oyster beds in the Maritime provinces peaked at over 175,000 t (whole animal weight; Bastien‐Daigle et al. 2007; Abgrall et al. 2010). The decline in oysters on natural beds prompted multiple transfers of Malpeque‐resistant individuals from Bedeque Bay, Prince Edward Island (PEI), to various river systems within the Maritime provinces (Logie 1958; Drinnan and England 1965; Drinnan 1967).

While these restoration efforts proved successful, little was known about the potential degree of genetic introgression between the ancestral (remnants) and introduced (Malpeque‐resistant) populations in those restored beds. It was hypothesized that re‐seeding efforts led to the homogenization of oyster populations throughout the Gulf of St. Lawrence by altering population structure from genetically distinct estuarine populations prior to the Malpeque disease outbreak to a more genetically homogeneous population following large‐scale translocations post‐outbreak. However, recent research has suggested that ancestral populations may have survived in some areas of the Maritime provinces (Vercaemer et al. 2010; Bernatchez et al. 2018). Using double‐digest Restriction site Associated DNA Sequencing (ddRAD‐seq) technologies, Bernatchez et al. (2018) found genetic differentiation among oysters in estuaries in the southern Gulf of St. Lawrence across New Brunswick (NB), Nova Scotia (NS), and PEI. In the Bras d'Or Lakes, NS, where the importation of oysters has been mainly prohibited since the onset of Malpeque disease, work by Vercaemer et al. (2010) using microsatellite DNA markers revealed that these oysters are genetically distinct from all other oysters originating from the Maritime provinces. Furthermore, historical literature indicates that salinity could act as a barrier to Malpeque disease transmission, suggesting that ‘small relict populations’ existed in areas of brackish water at the heads of bays and estuaries (Drinnan 1967). While genetic differentiation of oysters between waterbodies has been demonstrated (Vercaemer et al. 2010; Bernatchez et al. 2018), fine‐scale analysis of upriver populations in low‐salinity headwaters has not been assessed.

Evaluating and accounting for wild population genetics is essential for the sustainable management of both the aquaculture industry and emerging restoration projects. Within the growing oyster aquaculture industry, transfers of wild‐caught seed between river systems have been a common practice in eastern Canada, and internationally (McKindsey et al. 2007; Forrest et al. 2009). Following the recent diagnosis of *Haplosporidium nelsoni* (MSX) and *Perkinsus marinus* (Dermo) in the Gulf of St. Lawrence and the resulting devastation of oyster beds in the Maritimes, oyster reef restoration is beginning to be actively explored in eastern Canada (Clements et al. 2026). Oyster restoration is already widely implemented across other jurisdictions, including a number of tributaries in the United States (US), Australia, New Zealand, Europe and Asia, using both wild and cultured oysters and primarily serve as a management strategy for climate change adaptation (Southworth 1998; Airoldi et al. 2021; Vozzo et al. 2021; Howie and Bishop 2021). In addition, with these new detections of MSX and Dermo in the Maritime provinces, urgent efforts have been made to obtain MSX‐resistant oyster seed, including the establishment of local breeding programs and the transfer of US oysters into eastern Canadian waters (Atlantic Canada Opportunities Agency 2026).

Because oyster aquaculture often involves selecting specific seedstock, transferring cultured oysters may influence the genetic composition of wild populations inhabiting natural beds in areas where oyster aquaculture is widespread (Jaris et al. 2019). Given these well‐intended restoration efforts and urgent desires to acquire disease‐resistant seed, there is a need to understand the potential genetic impacts on wild oyster populations (Arnaud‐Haond et al. 2004; Camara and Vadopalas 2009; Hornick and Plough 2019). From an ecological perspective, altering the genetic structure of a native population can have unintended consequences. For instance, non‐local stocks may not be adapted to local environmental conditions and could carry or introduce pathogens or strains of pathogens to which native beds are susceptible (Wolff 2005; McKindsey et al. 2006; Camara and Vadopalas 2009). Because genetic diversity and population structure underpin a species' capacity to evolve and adapt to environmental change, an effort should be made to maintain the genetic integrity of wild oyster populations by using local seed whenever possible (Grant et al. 2017).

Building on knowledge gained in previous research (i.e., Bernatchez et al. 2018; Vercaemer et al. 2010), the present study aimed to analyse the genetic diversity and population structure of eastern oysters across oyster beds in NB, PEI and NS using ddRAD‐seq (Peterson et al. 2012). This study also examined the genetic diversity of oysters along estuarine gradients to understand if upriver and downriver oysters are genetically differentiated and to determine if ancestral populations persist in low‐salinity upriver locations. We hypothesized that: 1. Localized genetic structure between bays would be consistent with Vercaemer et al. (2010) and Bernatchez et al. (2018), largely owing to geographic variation in the degree and success of historical supplementation efforts; and 2. Within‐system genetic differentiation between up‐ and downriver oysters would be evident in some bays, reflecting the persistence of small relict populations. Ultimately, these results provide insight into fine‐scale genetic differentiation among oyster populations that may have been sheltered from the dramatic population declines caused by the emergence of Malpeque disease, offering valuable genetic baseline data to help inform ongoing restoration strategies and guide discussions surrounding seed transfer practices in eastern Canada.

## 2 Methods

### 2.1 Sites and Sample Collection

Between October 15, 2019, and November 9, 2020, a total of 1450 oysters were collected from wild natural beds across 25 locations in eastern Canada and the United States (Table 1). An additional 47 oysters from two sites in the Rappahannock River (Chesapeake Bay) were obtained through archived material stored in 95% Ethanol (collection date: November 22, 2010; National Aquatic Animal Health Laboratory, DFO Gulf Fisheries Center, Moncton, NB), resulting in a total of 1497 individuals (27 locations). Within 18 locations sampled in the Maritimes, there were nine areas that could be considered the same river system, with samplings occurring in upriver and downriver components (Table 1; Figure 1). All locations have been previously affected by Malpeque disease, except the Bras D'Or Lakes (Cape Breton) and USA, where the disease has not been reported. Oysters were collected by boat using either a drag, tongs, or rake, or handpicked on foot. Oysters were shipped to the Fisheries and Oceans Canada Gulf Fisheries Centre (Moncton, NB) on ice within 48–76 h of collection and kept refrigerated for up to a week before processing. Oysters were shucked with sterile instruments and live tissue was placed in 95% ethanol prior to DNA extraction. Surface water temperature at the time of collection varied between ~0.5°C and 21°C (Table 1). Water depth at collection sites ranged between 0.5 and 10 m.

**TABLE 1:** Details of oyster sampling locations. Temperature and salinity represent surface water measurements at the time of sampling.

Location	Location code	Up‐down river	Latitude	Longitude	Temp (°C)	Salinity (psu)	*n*
Nova Scotia							
Aspy Bay (north)	ASPY	—	46.90852	−60.51112	21.3	24.9	60
Arichat Harbour	ARI	Down	45.50497	−60.95965	7.1	20	60
Bras d'Or Lakes (East River)	BRASDOR_U	Up	45.91336	−61.05807	3.4	2.8	44
Bras d'Or Lakes (East River)	BRASDOR_D	Down	45.89867	−61.08033	10	14	57
Pictou	PICT_U	Up	45.6577	−62.77807	13	24.5	60
Pictou	PICT_D	Down	45.65736	−62.6895	13.5	24.9	60
New Brunswick							
Bouctouche	BOU_U	Up	46.41577	−64.81472	8.5	10	60
Bouctouche	BOU_D	Down	46.50594	−64.6563	8	25	60
Caraquet	CARA_U	Up	47.76833	−65.08486	8	12	60
Caraquet	CARA_D	Down	47.78249	−65.04262	10.5	25	60
Miramichi (Bartibog)	BART	—	47.08695	−65.36027	0.9	10	60
Miramichi (Bay du Vin)	VIN_U	Up	47.03041	−65.13593	5.4	3.6	60
Miramichi (Bay du Vin)	VIN_D	Down	47.0453	−65.14492	6.6	7.9	60
Miramichi (Loggieville)	LOGI	—	47.07387	−65.38344	11.4	15.6	60
Miramichi (Middle Island)	MMID	—	47.05069	−65.4563	8.1	4.3	60
Richibucto (Site 1287)	RICH_1287	Up (a)	46.58216	−64.94881	9.3	7.3	48
Richibucto (Site 1289)	RICH_1289	Up (b)	46.61736	−64.91875	9.7	10.3	60
Richibucto (Site 1293)	RICH_1293	Down	46.69258	−64.79275	8.6	20.7	60
Prince Edward Island							
Brudenell River	BRU_U	Up	46.19951	−62.61953	6.5	17.3	60
Brudenell River	BRU_D	Down	46.18186	−62.5398	3.6	26	60
Foxley River	FOX_U	Up	46.6759	−64.12221	9.5	6.5	60
Foxley River	FOX_D	Down	46.69335	−64.09893	10.9	25.2	60
Grand River	GR_U	Up	46.45619	−63.97821	7.3	3.1	60
Grand River	GR_D	Down	46.47897	−63.94988	12.4	24.1	60
United States							
Maine (Sheepscot River)	MAINE	Down	—	—	—	—	41
Chesapeake Bay (Rappahannock River)	CHESA1	—	—	—	—	—	25
Chesapeake Bay (Rappahannock River)	CHESA2	—	—	—	—	—	22

*Note:* *n* = number of oysters sampled.

**FIGURE 1:**
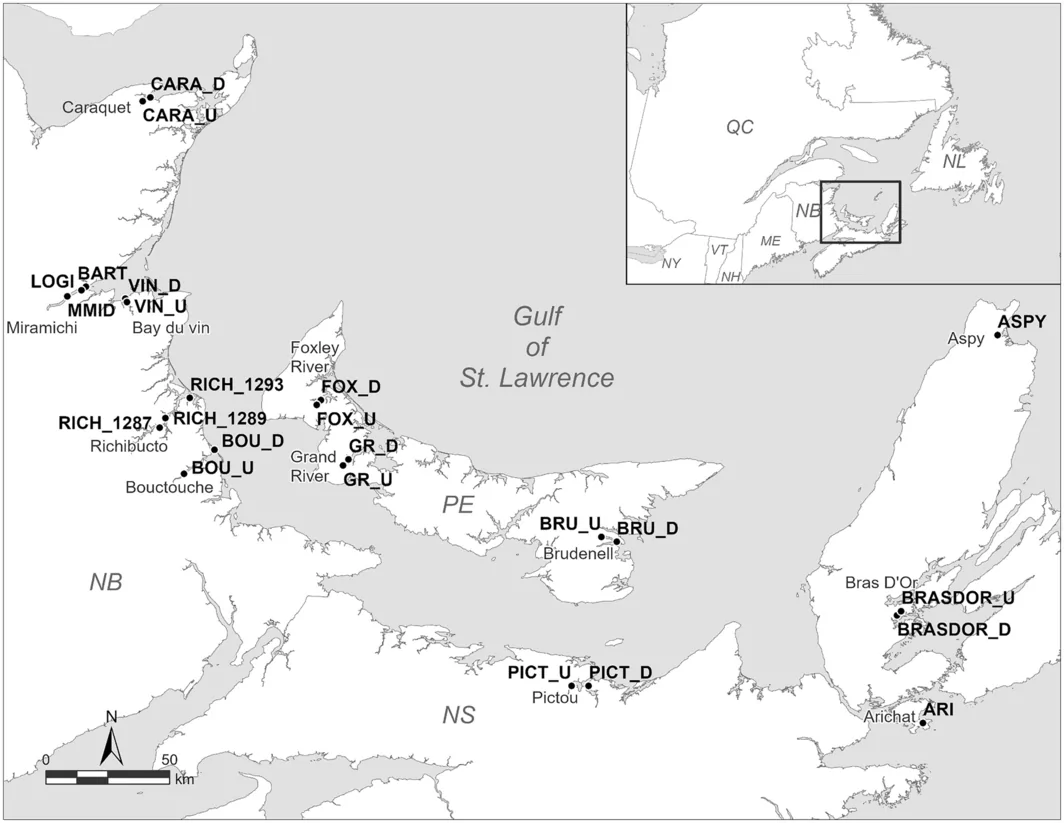
Map of oyster sampling locations in Canadian Maritime provinces. Map was generated using freely accessible shapefiles from the Global Administrative Areas (GADM) database (https://gadm.org/license.html).

### 2.2 DNA Extraction, Library Preparation and Sequencing

Ethanol preserved adductor muscle tissue was used for DNA extractions. Each oyster's DNA, with the exception of Aspy River, was extracted using a KingFisher Flex instrument (Thermo Fisher Scientific, Waltham, MA), using a MagMAX Core nucleic acid purification kit (Thermo Fisher Scientific, Waltham, MA) following manufacturer instructions. Aspy River was extracted using a homemade DNA extraction kit following the protocol from (Ivanova et al. 2006). DNA was sent to the University of Laval, Quebec for library preparation. The restriction enzymes Nsil and MspI were used to digest and fragment DNA. Libraries were sent to Genome Quebec, Montreal, QC, for Illumina NovaSeq 2500 M 150 bp paired‐end sequencing.

### 2.3 ddRAD‐Seq Analysis

Raw ddRAD‐seq data was processed using Stacks v2.55 (Catchen et al. 2013; Rochette et al. 2019). RAD‐tags were demultiplexed and underwent quality control and filtering using process radtags, in paired‐end mode, with adaptors being trimmed and two mismatches allowed. Retained RAD‐tags of each oyster sample were aligned to the *C. virginica* genome (GCF_002022765.2) using BWA‐mem in paired‐end mode (Li 2013). A catalogue of loci was assembled in Stacks g‐stacks pipeline (Catchen et al. 2013; Rochette et al. 2019). The population pipeline in Stacks was used to identify a set of SNP loci given a population map. In total, ten population maps were analysed in the population pipeline. The first population map was used to analyse genetic diversity between all locations within the Maritime provinces described in Table 1. The remaining nine population maps analysed genetic diversity along estuarine gradients within each location. Each location with upriver‐downriver counterparts was analysed separately (Table 1). Within the population pipeline, loci were filtered by minimum percentage of individuals in a population of < 65%. SNPs were further filtered using PLINK v1.90b (Purcell et al. 2007; Slifer 2018). SNP missingness was analysed with oysters being filtered out if missing > 60% of all SNP loci. SNP loci were also filtered out if missing from 10% of individuals, and minor allele frequency of < 5%. Effective population sizes *N*_*e*_ were estimated for each location using the single‐sample Linkage Disequilibrium *LDN*_*e*_ method (Waples 2006) performed using NeEstimator v2.1 (Do et al. 2014). Linkage disequilibrium was calculated in PLINK, with linked SNP loci at *r* ^2^ ≥ 0.1 being removed from the dataset. Summary statistics including heterozygosity (*H*_*o*_ and *H*_*e*_), fixation index (FST), inbreeding coefficient (FIS), and nucleotide diversity (*Pi*) were computed using the population pipeline in Stacks, PLINK, and in VCFtools (Danecek et al. 2011).

### 2.4 SNP Loci Putatively Under Selection

Filtered SNP loci were analysed to identify loci as putatively neutral or under diversifying selection. To identify SNP loci that were neutral or divergent between oyster locations and estuarine gradients, two methods were used: (1) an island model using Arlequin v.3.5 (Excoffier and Lischer 2010); and (2) Bayesian method using BayeScan v. 2.1 (Foll and Gaggiotti 2008). In Arlequin, analysis was run using 200,000 simulations and 1000 demes. In BayeScan, the analysis was run using a burn‐in period of 200,000, prior odds of 10,000, and 10,000 iterations. The results of both analyses resulted in the classification of SNP loci considered neutral or under diversifying selection. In Arlequin, SNPs were considered under selection if they had a *p*‐value ≤ 0.05 and observed FST ≥ 0.1. In BayeScan, SNP loci were considered under selection if they had a *q*‐value ≤ 0.05. All SNP loci identified as under selection were pooled to have one group of SNP loci under selection per analysis completed. The neutral SNP loci group consisted of all SNPs not found under‐selection by Arlequin or Bayescan, with balancing SNPs (α < 0) identified by BayeScan removed.

### 2.5 Population Structure and Assignment

SNP loci datasets of population structure between locations and estuarine gradients were analysed for genetic structure using several methods. SNP loci datasets were analysed in ADMIXTURE v1.3.0 (Tang et al. 2005; Alexander and Lange 2011) to determine the most likely number of ancestral populations (*K*) of a given dataset. The number of *K* was determined by computing cross‐validation (CV) error with *K =* 1–8, to determine the lowest CV error compared to all other *K* values. SNP loci datasets were also analysed by computing principal component analysis (PCA) in PLINK (Purcell et al. 2007; Slifer 2018) and PCA was visualized in the R package ggplot2 (Wickham 2016). Genetic distance of SNP loci datasets was computed in poppr v.2.6.1 (Kamvar et al. 2014, 2015) and ape v.5.0 (Paradis and Schliep 2019), to compute Provesti's distance between oyster locations and visualize an UPGMA dendrogram. Results of population structure analysis were used to compute an analysis of molecular variance (AMOVA) to understand variance within oysters, between oysters, between oyster location, and among population structure groupings using R packages adegenet v.2.1.8 (Jombart and Bateman 2008) and poppr v.2.6.1 (Kamvar et al. 2014, 2015). SNP loci datasets were analysed using all loci to determine if differentiation existed between locations and estuarine gradient.

### 2.6 Isolation by Distance

Isolation by distance (IBD) was assessed among the sampling locations across the Maritime provinces using filtered, linkage disequilibrium‐pruned SNP dataset. Pairwise genetic differentiation was estimated using Weir and Cockerham's (1984) *F*_st_, transformed into linearized genetic distances following Rousset (1997) $\left[F_{\mathrm{ST}}/\left(1-F_{\mathrm{ST}}\right)\right]$. Geographic distance matrices were generated from GPS coordinates for each sampling locality, with pairwise distances (km) calculated using the great‐circle Haversine formula implemented in the R package geosphere v1.5–18 (Hijmans 2023). The relationship between the linearized genetic and geographic distance matrices was evaluated using a Mantel test (Mantel 1967) with Pearson's correlation coefficient in the R package vegan v2.6–4 (Oksanen et al. 2024) using 9999 matrix permutations to determine statistical significance. To evaluate whether the Bras d'Or Lake populations exerted a disproportionate influence on spatial structure, two separate IBD analyses were conducted: an initial analysis incorporating all sampling locations across the Maritime provinces, followed by a secondary analysis excluding the Bras d'Or Lake populations.

### 2.7 SNP Loci Annotation

To identify potential gene identities and infer potential biological functions, SNP loci putatively found under selection were functionally annotated from each analysis completed. These outlier loci were mapped to the *Crassostrea virginica* reference genome (NCBI Assembly Accession: GCA_002022765.4) using a BLASTn alignment search and characterised using corresponding GFF3 structural annotation features. To assign Gene Ontology (GO) terms, the RefSeq protein accessions associated with the annotated SNP coordinates were extracted and queried against the UniProt database. Due to the limited number of mapped candidate loci, formal statistical over‐representation tests were not performed. Instead, for each set of SNP loci putatively under selection, a descriptive measure of GO term distribution percentage was calculated by determining the percentage of unique SNP loci mapped to a specific GO term relative to the total number of unique SNP loci within a particular comparison.

## 3 Results

### 3.1 Populations and Summary Statistics

Initially the analysis was computed including USA oyster locations; however, these samples exhibited a high level of SNP missingness during initial filtration steps. Furthermore, initial principal component analysis and ADMIXTURE clustering of individuals surviving initial filtration demonstrated that there was genetic divergence between Maritime oysters and those from the USA (Chesapeake Bay and Maine), which obscured the more subtle population structure among the Maritime province estuaries (Figures S1–S3 in File S1). Consequently, the USA locations were excluded from all subsequent downstream analysis to focus on regional estuarine dynamics.

Initially 2,803,814 SNP loci were identified within g‐stacks catalogue; however, after completing the population pipeline and filtering steps using ten population maps to identify genetic diversity of locations within the Maritimes, and estuary analysis, the final number of SNP loci was between 14,977 and 21,477 (Table 2). A total of 1385 oysters were retained after quality filtering. Effective population size (*N*_*e*_) was found to range from 973.3–3924.2 (Table 3). Observed heterozygosity ranged between 0.176–0.189 and was lower than expected heterozygosity, which ranged from 0.236 to 0.238 (Table 3). At all locations, FIS was positive and ranged from 0.201 to 0.241 (Table 3). Nucleotide diversity ranged from 4.96E‐03 to 7.82E‐03, with NS oyster locations containing lower nucleotide diversity compared to NB and PEI. Effective population size estimates ranged from 973.3 to 3924.2. Average pairwise FST varied by analysis, with the genetic diversity of oysters between locations having a higher average FST (0.012) compared to the analysis of oysters between estuarine gradients (BRASDOR_D/U: 0.003; VIN_D/U: 0.003; RICH_1287/1289/1293: 0.003; CARA_U/D: 0.002; BOU_D/U: 0.003; BRU_D/U: 0.003; PICT_D/U: 0.002; GR_D/U: 0.003; FOX_D/U: 0.003).

**TABLE 2:** Number of SNP loci present initially and after each filtering step in each of the groups analysed in this study.

Analysis	Location code	SNPs after populations	SNPs after PLINK	LD pruned (*r* ^2^ ≥ 0.1)
(1) Diversity between locations	All Maritimes locations	2,061,976	61,607	15,912
(2) Diversity of oysters along an estuarine gradient	BRASDOR_D, BRASDOR_U	680,071	112,682	14,977
	VIN_D, VIN_U	909,867	110,593	17,698
	RICH_1287, RICH_1289, RICH_1293	872,145	102,685	17,541
	CARA_D, CARA_U	964,716	96,031	20,121
	BOU_D, BOU_U	924,679	102,310	19,187
	BRU_D, BRU_U	848,386	83,909	18,251
	PICT_D, PICT_U	862,799	99,651	18,570
	GR_D, GR_U	997,114	107,359	21,542
	FOX_D, FOX_U	1,042,748	107,429	21,477

**TABLE 3:** Overview of summary statistics calculated for each location using the analysis of genetic diversity between all locations SNP loci dataset.

Cluster	Location code	*Ho*	*He*	*Fis*	*Pi*	*Ne*
Cluster 1 (‘Gulf’ region)	GR_D	0.184	0.237	0.231	7.53E‐03	2390.7
	GR_U	0.183	0.238	0.224	7.71E‐03	3579.0
	FOX_D	0.186	0.237	0.223	7.45E‐03	2290.0
	FOX_U	0.184	0.237	0.214	7.58E‐03	2693.1
	CARA_D	0.184	0.237	0.223	7.40E‐03	2172.1
	CARA_U	0.186	0.237	0.215	7.61E‐03	2482.0
	BRU_D	0.179	0.236	0.242	7.57E‐03	973.3
	BRU_U	0.183	0.237	0.231	7.82E‐03	3846.3
	PICT_D	0.187	0.237	0.210	4.96E‐03	1857.5
	PICT_U	0.189	0.236	0.201	5.07E‐03	1377.3
	BOU_D	0.180	0.237	0.241	7.21E‐03	2993.8
	BOU_U	0.186	0.238	0.218	7.63E‐03	3343.2
Cluster 2 (Miramichi region)	MMID	0.186	0.237	0.217	7.12E‐03	2164.0
	BART	0.184	0.237	0.222	6.99E‐03	2124.4
	VIN_D	0.185	0.238	0.222	7.30E‐03	3790.7
	VIN_U	0.185	0.237	0.220	7.57E‐03	3355.5
	LOGI	0.189	0.238	0.205	7.32E‐03	3924.2
Cluster 3 (Coastal Cape Breton)	ASPY	0.180	0.237	0.239	5.42E‐03	1224.8
	ARI	0.186	0.237	0.216	7.33E‐03	1721.9
Cluster 4 (Richibucto region)	RICH_1293	0.185	0.238	0.207	7.55E‐03	1879.9
	RICH_1289	0.187	0.237	0.213	7.39E‐03	2152.9
	RICH_1287	0.189	0.238	0.220	7.28E‐03	2571.6
Cluster 5 (Bras d'Or Lakes region)	BRASDOR_D	0.176	0.238	0.259	6.96E‐03	2852.6
	BRASDOR_U	0.177	0.238	0.255	6.94E‐03	2741.9

### 3.2 SNP Loci Putatively Under Selection

Across all analyses completed in this study (i.e., the overall analysis, as well as the estuarine gradient analysis), SNP loci were found putatively under selection and candidate SNP loci were distributed among all chromosomes, suggesting that putative signatures of selection were not restricted to specific genomic regions. The overall analysis including all sampling locations across the Maritime provinces identified the greatest number of SNP loci putatively under selection, with 312 loci detected by BayeScan and 39 loci detected by Arlequin. Of these, 31 SNP loci were identified by both approaches (Table 4; see File S2 for the complete list of candidate loci). In comparison, fewer putatively selected SNP loci were detected within the individual estuarine gradient analyses (Table 4; File S2); however, each estuarine analysis identified a set of divergent SNP loci distinguishing upriver and downriver sampling locations. Comparison of candidate loci identified among estuarine gradient analyses revealed 12 SNP loci that were shared between at least two estuarine systems. These shared loci included five SNPs detected between BOU_D/U and GR_D/U, two between GR_D/U and VIN_D/U, and one each between BOU_D/U and VIN_U/D, CARA_D/U and RICH_1287/1289/1293, GR_D/U and FOX_D/U, BOU_D/U and FOX_D/U, and PICT_D/U and BRU_D/U.

**TABLE 4:** Overview of the number of SNP loci under selection (S), neutral (N), and balancing (B) using Arlequin and BayeScan for each analysis completed in this study. The number of SNP loci significantly under selection overlapping mutually between the two software is also displayed.

Analysis	Location code	Arlequin	BayeScan	SNPs found under selection in both software
(1) Diversity between locations	All Maritimes locations	S: 39; N: 15,873	S: 312; N: 11,393; B: 4207	31
(2) Diversity of oysters along an estuarine gradient	BRASDOR_D, BRASDOR_U	S: 39; N: 14977	S: 0; N: 11,793; B: 3224	0
	VIN_D, VIN_U	S: 46; N: 17,652	S: 2; N: 13,874; B:3822	1
	RICH_1287, RICH_1289, RICH_1293	S: 28; N: 17,533	S: 3; N: 13,732; B: 3804	3
	CARA_D, CARA_U	S: 32; N: 20,089	S: 1; N: 15,920; B: 4200	1
	BOU_D, BOU_U	S: 44; N: 19,121	S: 2; N: 17,336; B: 4602	2
	BRU_D, BRU_U	S: 35; N: 18,216	S: 0; N: 14,392; B: 3859	0
	PICT_D, PICT_U	S: 38; N: 18,532	S: 0; N: 14,614; B: 3956	0
	GR_D, GR_U	S: 66; N: 21,411	S: 3; N: 14,590; B: 6884	3
	FOX_D, FOX_U	S: 54; N: 21,488	S: 2; N: 16,875; B: 4665	2

Abbreviations: B, Balancing SNP loci (only in BayeScan); N, Neutral SNP loci; S, SNP loci under selection.

### 3.3 Population Structure and Assignment

The analysis of all locations to understand genetic diversity between locations in the Maritime provinces found differentiation using ADMIXTURE, PCA and genetic distance analysis between SNP loci datasets (Figures 2 and 3). ADMIXTURE analysis identified *K* = 4 as the optimal number of ancestral populations based on the lowest cross‐validation (CV) error (see Figure S4 in File S1 for ADMIXTURE *K = 1–8* CV error), and this value was used for subsequent visualization (Figure 2A). The ADMIXTURE plot revealed four major ancestral components. One cluster (Cluster 1) was generally widespread among multiple sampling locations in the Gulf of St. Lawrence, including NB (BOU_D, BOU_U, CARA_D, and CARA_U), PEI (FOX_D, FOX_U, GR_D, GR_U, BRU_D, and BRU_U), and NS (PICTOU_D and PICTOU_U), and was designated the ‘Gulf’ cluster. Other clusters represented the Miramichi, NB region (BART, LOGI, MMID, VIN_D, and VIN_U; Cluster 2), the Richibucto, NB region (RICH_1287, RICH_1289, and RICH_1293; Cluster 4), and the Bras d'Or Lakes region (BRASDOR_U and BRASDOR_D; Cluster 5). In contrast, while individuals from ASPY and ARI clustered (Cluster 3), they did not exhibit a distinct dominant ancestral component but instead displayed mixed ancestry profiles, primarily consisting of the Gulf (Cluster 1) and Bras d'Or Lakes (Cluster 5) ancestral components. These populations were collectively termed the ‘Coastal Cape Breton’ group and exhibited genetic characteristics intermediate between the Gulf (Cluster 1) and Bras d'Or Lakes (Cluster 5) clusters. Developed dendrograms (Figure 2b) also agreed with this clustering. In the dendrograms, some separation patterns could be visualized within the ‘Gulf’ cluster, indicating some differentiation among this large cluster (Figure 2b). PCA analysis (Figure 3) also followed similar visualization patterns as ADMIXTURE and Dendogram visualization. An AMOVA was completed using the four ancestries identified in ADMIXTURE. Results of the AMOVA for each SNP loci dataset are displayed in Table S1 in File S1. Variation within oyster samples, between oyster samples, between oyster locations, and among population structure groupings were all found significant in each loci dataset examined; however, the level of variation differed between SNP loci datasets.

**FIGURE 2:**
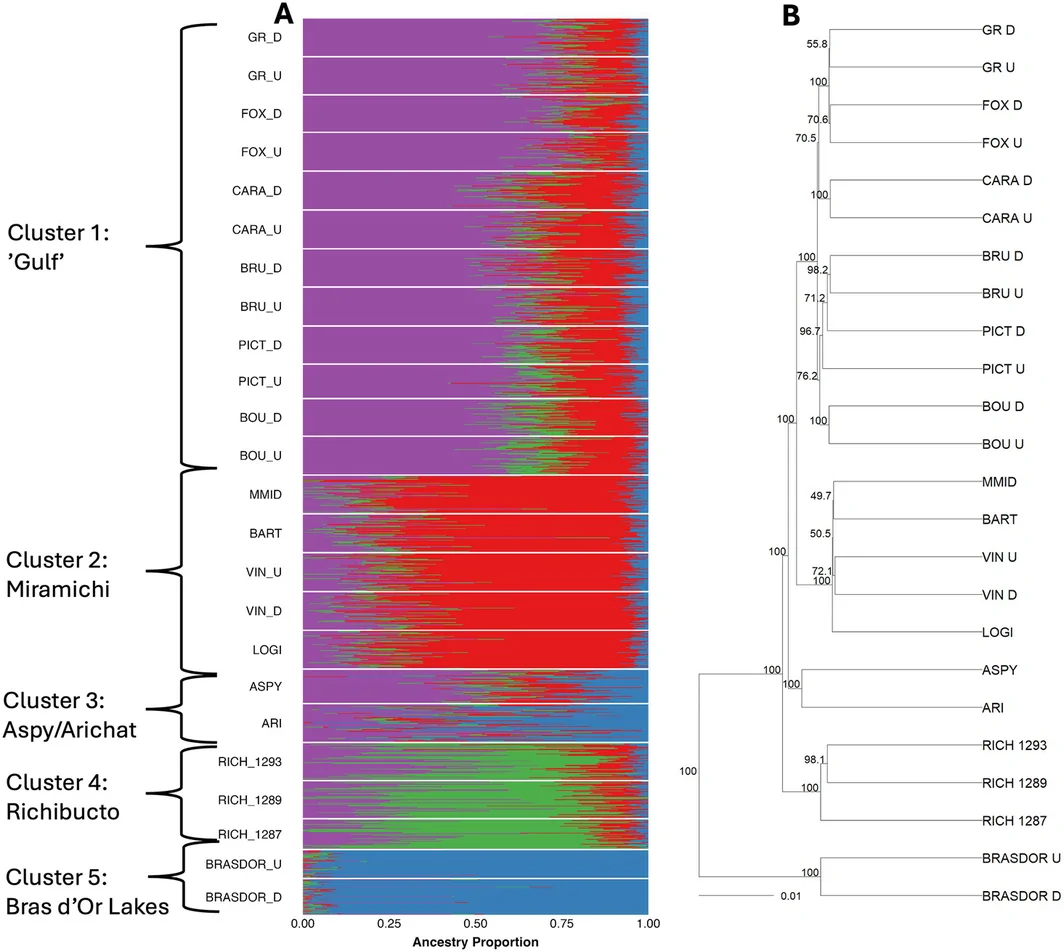
(A) ADMIXTURE plot showing population genetic structure at *K* = 4 and (B) UPGMA dendrogram based on Prevosti's genetic distance for 24 oyster sampling locations across the Maritime provinces. Both analyses were conducted using the filtered SNP dataset containing 15,912 SNP loci.

**FIGURE 3:**
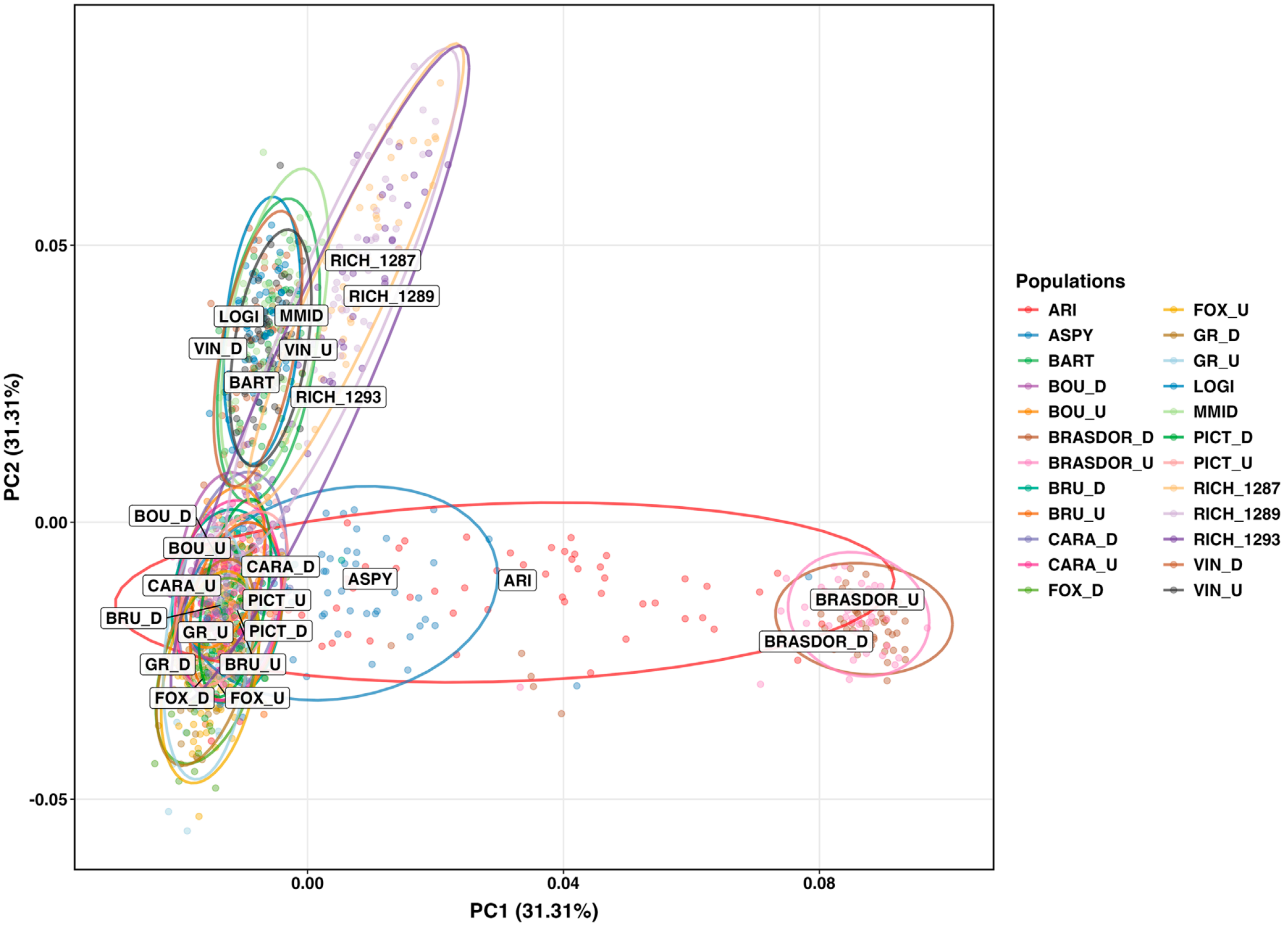
Principal component analysis (PCA) illustrating patterns of genetic variation among individuals from 24 oyster sampling locations across the Maritime provinces. The PCA was generated using the filtered SNP dataset consisting of 15,912 SNP loci.

Despite finding SNP loci under selection, analysis of estuary gradients did not find differentiation between upriver/downriver locations. Analysis in ADMIXTURE using all SNP loci found that the *K* with the lowest CV error was *K =* 1 in all estuary analyses, indicating that each estuary was not significantly differentiated. Similarly, PCA analyses using all SNP loci (Figure 4) did not show differentiation; however, when examining PCA using SNPs loci under selection (Figure 5), differentiation between the estuarine gradients was apparent.

**FIGURE 4:**
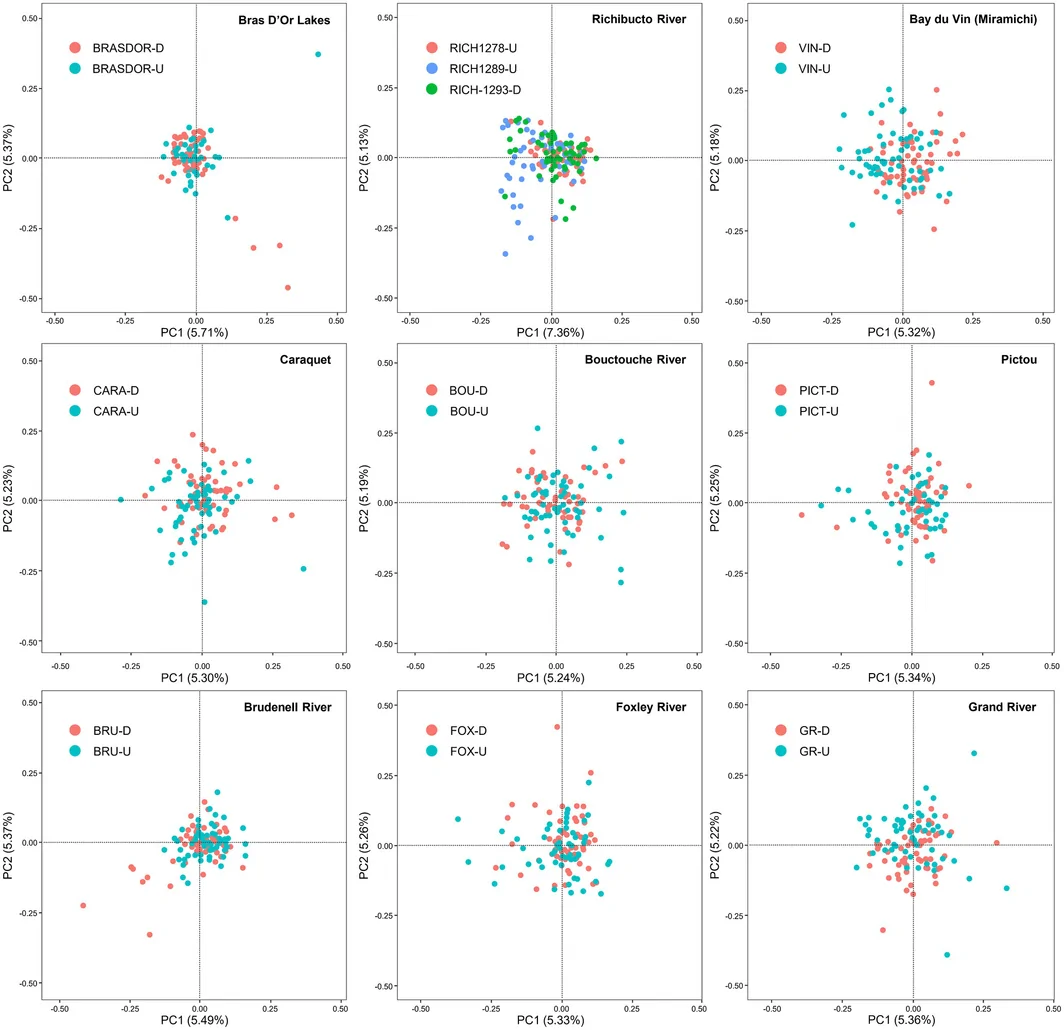
Principal component analysis (PCA) illustrating patterns of genetic variation among individuals from paired upriver and downriver oyster populations across estuarine gradients. PCAs were generated using filtered SNP datasets (Table 2) containing 14,977–21,542 loci, depending on the location comparison.

**FIGURE 5:**
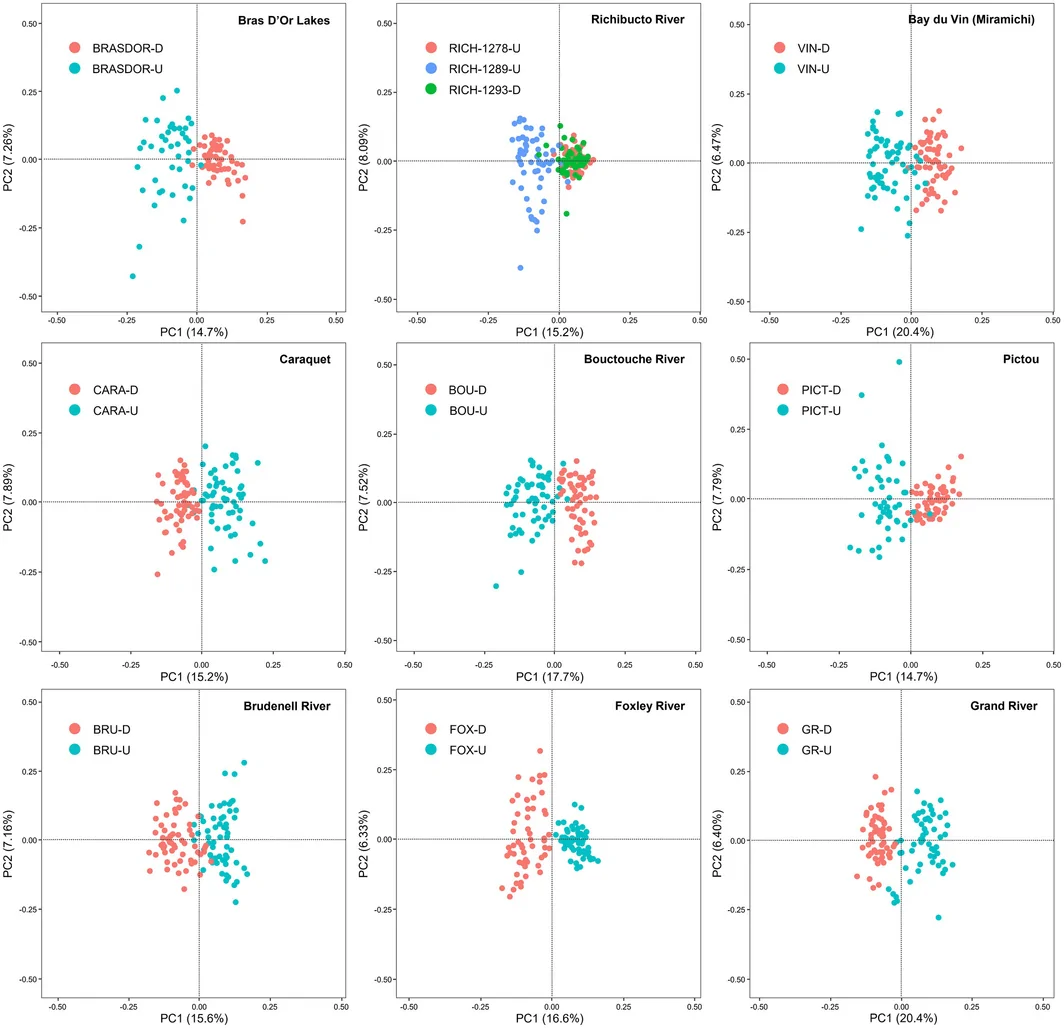
Principal component analysis (PCA) illustrating patterns of genetic variation among individuals from paired upriver and downriver sampling locations across estuarine gradients using SNP loci putatively under selection. PCAs were generated using SNP loci identified as under selection (Table 3) containing 28–66 loci, depending on the location comparison.

### 3.4 Isolation by Distance

A Mantel test revealed that geographic distance was a significant driver of genetic differentiation across the study area (*p* = 0.0020), exhibiting a moderate positive linear relationship (*R* ^2^ = 0.4462). Figure 6A illustrates this relationship using the pairwise genetic and geographic distances calculated among all 24 sampling locations (see Table S2 in File S1 for the complete linearized matrix). However, despite this overarching isolation by distance (IBD) trend, the relationship was highly non‐uniform, with samples from the Bras d'or Lakes populations skewing the relationship. A second Mantel test was performed, removing the Bras d'or Lakes populations (Figure 6B), revealing that geographic distance remained a significant predictor of genetic structure (*p* = 0.028), with a moderately positive linear relationship (*R* ^2^ = 0.261).

**FIGURE 6:**
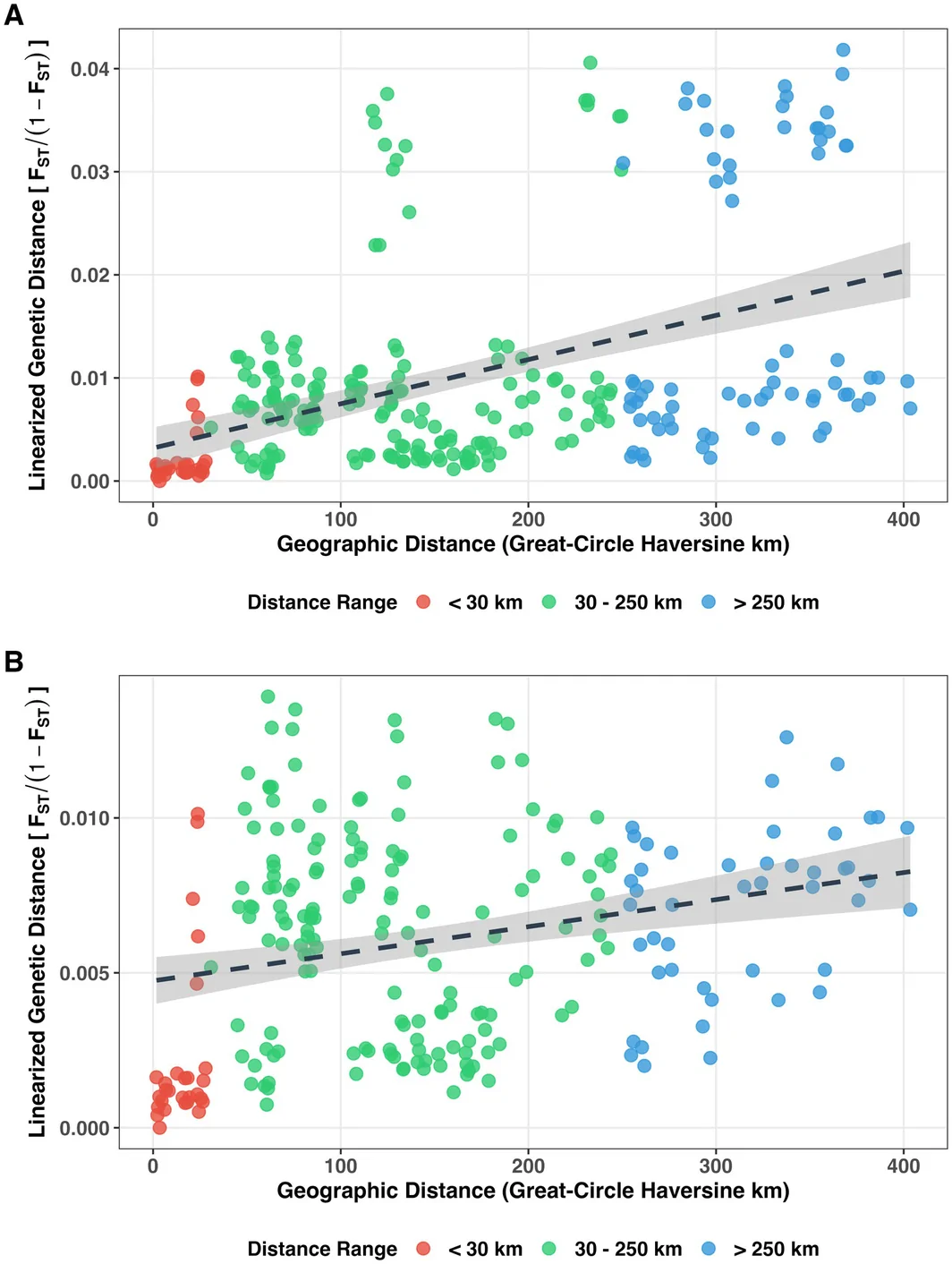
Isolation by distance (IBD) plots showing the relationship between pairwise geographic distance (km) and genetic distance *F*_ST_/(1−*F*_ST_) for the IBD analysis across 24 sampling locations (A), and the IBD analysis with BRASDOR populations removed (B). Data points are colour‐coded as a function of geographic distance between pairwise comparisons.

### 3.5 SNP Loci Annotation

A full description of the SNP loci under selection for each analysis and associated *C. virginica* gene and annotation can be found in File S3. Within the analyses evaluating estuarine gradients, 12 SNP found to be under selection were shared mutually among multiple independent systems, four of which were successfully annotated: putative aminopeptidase (Bouctouche and Grand River), circularly permuted Ras protein 1‐like (Bouctouche and Grand River), transportin‐3‐like isoform X1 (Pictou and Brudenell River), and origin recognition complex subunit 1 (Bouctouche and Bay du Vin).

The functional profiles derived from the descriptive review of GO terms are summarized in File S4. Specifically, SNP loci under selection were annotated to genes with GO terms heavily concentrated within functions relating to membrane and cellular components, protein functions, signalling networks, and ion binding processes. Within the analysis evaluating estuarine gradients, common GO term functional groups were found to be primarily associated with protein degradation and modification cascades, structural cellular compartmentalization, localized macromolecular metabolism and cell division, and transport and ion‐binding pathways (File S4).

## 4 Discussion

In this study, we were interested to see how much the large‐scale transfers that occurred in the 1930s and 1960s to restore depleted oyster stocks from the Malpeque disease epidemic impacted the genetic diversity of oysters in the Maritimes provinces. Recent research has examined this (Vercaemer et al. 2010; Bernatchez et al. 2018) and the present study aimed to build on this research by further analysing the genetic diversity between wild oyster beds from NB, PEI, and NS, and to further understand if there was genetic structure between upriver–downriver oyster beds.

### 4.1 Genetic Diversity of Oysters Between Oyster Beds in the Maritimes

The present study agrees with previous research showing that there is genetic diversity between oyster beds in the Maritime provinces (Vercaemer et al. 2010; Bernatchez et al. 2018). Analysis of genetic structure in ADMIXTURE found that the number of ancestral populations (*K*) with the lowest level of error was four. Differentiation in ADMIXTURE demonstrated that a large cluster (Cluster 1) emerged with similar ancestral composition containing oysters from PEI (Brudenell River, Foxley River, Grand River), NB (Bouctouche, Caraquet), and NS (Pictou). In addition, oysters in the Miramichi (Cluster 2), Richibucto (Cluster 4), and Bras d'Or Lakes (Cluster 5) regions all appeared to have different dominant ancestral lineages. Coastal Cape Breton locations (Aspy River and Arichat; Cluster 3) were similar in genetic composition, containing a mixture of ancestral composition similar to both the Bras d'Or Lakes and the ‘Gulf’ clusters. Further analysis using PCA and dendrogram construction showed clustering similar to the ADMIXTURE analysis. Vercaemer et al. (2010) analysed the genetic diversity of oysters in the Maritime provinces using microsatellite analysis and found that oysters in the Cape Breton region displayed genetic differentiation, particularly in the Bras d'Or Lakes, which appeared to be highly differentiated. The present study also found that Cape Breton oysters contained genetic differentiation, with the Bras d'Or Lakes being differentiated from all other locations in the Maritimes. This finding is not unexpected as the Bras d'Or Lakes are semi‐enclosed (Taylor and Shaw 2002), which would limit natural genetic dispersion. The Bras d'Or Lakes are the only known area in the Maritimes that was not impacted by mortality associated with Malpeque disease (McGladdery and Bower 1999). However, they were the first region in the Maritimes where MSX was detected in 2002 (Stephenson et al. 2003). As a result of their unique disease history, oyster transfers into and out of the Bras d'Or Lakes have historically been prohibited.

Similar to the present study, Bernatchez et al. (2018) used a ddRAD‐seq approach with the same restriction enzymes to analyse genetic diversity between oyster beds in the Gulf of St. Lawrence. Summary statistics such as inbreeding coefficient (Fis = 0.201–0.241) and observed heterozygosity (Ho = 0.176–0.189) in our study were similar to levels reported by Bernatchez et al. (2018) (Fis = 0.188–0.211; Ho = 0.22–0.23). In addition, Bernatchez et al. (2018) reported relatively low *F*_st_ values (up to 0.0228) but significant population structure between oysters from different locations in the Gulf of St. Lawrence, including clusters that separated Caraquet, Miramichi, Richibucto, Bouctouche, and other locations within PEI and NS. Similarly, the present study also found a low mean *F*_st_ value (0.012) within the analysis between all locations, and genetic structure was also observed among oysters within the Gulf of St. Lawrence, with Miramichi and Richibucto oysters showing differentiation. In contrast, nucleotide diversity (*Pi* = 4.96E‐03–7.82E‐03) in this study was found to be larger than what was reported by Bernatchez et al. (2018). This is likely due to differences in calculation methodology between the two studies, specifically our focus on site‐level polymorphic variants rather than the haplotype‐wide averages deployed by Bernatchez et al. (2018). In addition, SNP missingness in our Aspy River location appeared to be higher than other locations included in the study (Figure S1 in File S1). Stringent filtering (65% site presence in Stacks, followed by a 10% maximum missingness and 5% minor allele frequency cutoff in PLINK) was employed specifically to maintain high data quality despite these sequencing challenges in the Aspy River cohort, allowing us to keep a reasonable number of individuals from this location while maintaining strict rigor in our SNP quality control.

### 4.2 Comparison of Genetic Diversity and Historical Transplants

In this study, an isolation by distance (IBD) analysis was conducted to evaluate spatial genetic structure across the region. The relationship was found to be statistically significant (*p* = 0.0020), exhibiting a positive linear relationship (*R* ^2^ = 0.4462). This trend was primarily driven by pairwise comparisons involving the highly isolated Bras d'Or Lakes, as well as comparisons between the Richibucto and Miramichi samples (Table S2 in File S1), which is consistent with the population genetic structure observed. However, despite this overall macro‐geographic trend, Figure 6 reveals a non‐uniform distribution. In an attempt to understand whether the Bras d'Or Lake populations exerted disproportionate influence on spatial structure, a second Mantel test was run excluding Bras d'Or Lakes populations. The second Mantel test revealed a more uniform distribution (Figure 6B) and demonstrated that geographic distance remained a significant predictor of genetic structure (*p* = 0.028) with a moderately positive linear relationship (*R* ^2^ = 0.261). However, several pairwise comparisons separated by vast geographic distances display low genetic distances comparable to sites in close proximity. This pattern is consistent with a more complex scenario: while physical distance influences regional genetic diversity, other anthropogenic and ecological factors may also be involved.

The ‘Gulf’ cluster (Cluster 1) is connected and likely derived from shared genetic ancestry. This is also consistent with historical oyster transfers that occurred after outbreaks of Malpeque disease. In 1915, Malpeque disease devastated Malpeque Bay and surrounding areas and resulted in mass mortality in the region (Found and Logie 1957). By 1935, outbreaks of the disease occurred in eastern PEI (Charlottetown Harbour‐Hillsborough Bay) (Found and Logie 1957), and oysters were transplanted from disease resistant areas with a goal to accelerate the recovery to around 10 years (Found and Logie 1957). The disease spread to NB and NS between 1952 and 1960, which prompted more oyster transfers from Bedeque Bay, PEI between 1956 and 1962 to NB and NS (ranging from Miscou, NB to Merigomish, NS) (Drinnan and England 1965; Drinnan 1967). During this time, ~11,000 barrels of oysters (equivalent to 5.5 million oysters) were transferred to all oyster producing regions impacted (Drinnan and England 1965; Drinnan 1967). Restoration efforts continued after the initial transfers occurred (Andrews 1971, 1972; Bacon 1979; Jenkins and Morrison 1997), including extensive experimentation to learn to collect wild oyster spat (Andrews 1971, 1972). Restoration efforts were particularly successful in Caraquet Bay and Bouctouche, with these areas becoming major seed producing regions and opening commercial operations in 1985 (Méthé and Léger 1994). Purchasing oyster seed from these regions became common practice in oyster aquaculture operations (Doiron 2008).

Despite the Miramichi and Richibucto regions receiving oyster transplants, they were both found to be genetically differentiated from the ‘Gulf’ cluster, a result consistent with Bernatchez et al. (2018). This genetic divergence may be explained in part by variable success rates of historical restoration efforts across different coastal systems as described by Bacon (1979). Bacon (1979) discussed the need to continue to reseed beds in New Brunswick, due to lack of rehabilitation of oyster stocks. This was attributed to the a lack of raking and tonging on leases, which helped to maintain oyster beds, following the shutdown of the oyster fisheries during the Malpeque disease epidemic. The act of raking or tonging on oyster beds helps indirectly with spat recruitment through shell desilting (Bacon 1979). In the Miramichi and Richibucto regions, it is not known how successful transplants were; however, in both areas there are reports of relay fisheries that move upriver oysters to leases located downriver (Andrews 1971; Jenkins and Morrison 1997). Experimental studies in the mid to late 1970s also found that oysters transferred from a PEI hatchery and from the Bouctouche area grew substantially slower in Bay du Vin and Baie Ste. Anne, than other areas of New Brunswick, which was attributed to lower salinity and colder temperatures from the Miramichi River runoff (Bacon 1979). Bacon (1979) pointed out, anecdotally, that other areas of the Miramichi had high quality oysters growing, and that efforts should be made to reseed rather than culture activities. Milewski and Chapman (2002) pointed out that oyster beds downriver were likely seeded naturally by upriver areas such as Chatham and Newcastle of the Miramichi River. This dynamic may be further explained by the Sweepstakes Reproductive Success (SRS) hypothesis, a phenomenon common in marine broadcast spawners like oysters (Hedgecock and Pudovkin 2011). Under SRS, volatile estuarine oceanography requires specific environmental conditions for successful larval survival, meaning only a fraction of adults successfully contribute to any given generation. If transplanted PEI oysters and offspring were less suited for environmental conditions in the Miramichi and Richibucto locations, their genetic contributions could be minimized. While this study did not directly track individual reproductive cohorts to analyse SRS and therefore can't definitively determine if this was factor, the effective population size (*N*_*e*_) was calculated across all locations, ranging from 973.3 to 3924.2 (Table 3), demonstrating that these beds maintained oyster population reservoirs. It is thus possible that native oyster beds in the Miramichi and Richibucto area were more successful at restoring themselves than the transplanted oysters from PEI.

This study also examined the genetic diversity of oysters along estuarine gradients to understand if upriver/downriver oysters may be genetically differentiated. Drinnan (1967) indicated that salinity could act as a barrier for disease transmission and reported that ‘small relict populations’ existed in areas of brackish water at heads of bays and estuaries (Drinnan 1967). The results of this study suggest that each upriver/downriver location within a system is likely not significantly genetically differentiated, with the results of ADMIXTURE reporting the number of ancestral populations with lowest error being one in each estuary. This was found in locations that had received historical oyster transplants, as well as locations such as the Bras d'Or Lakes where transplants did not occur. Clustering using all SNP loci in PCA analysis also did not show differentiation between upriver and downriver locations. Therefore, the results of this study suggest that genetic exchange may have occurred across the estuarine gradients of each river system resulting in a genetic similarity of oyster stocks. Relay fisheries were common practice after Malpeque disease in the Maritimes provinces, which may have had some influence (Jenkins and Morrison 1997), but the similarity of oyster stocks between upriver and downriver locations indicates that there may have been natural dispersal between the estuaries as well. Oyster larval dispersal trends in river systems depend on numerous factors including vertical migration of larvae, currents, tides, and mixing (Kennedy 1996).

### 4.3 Functional Characterization of SNP Loci Under Selection and Associated Evolutionary Mechanisms

The present study identified 320 candidate SNP loci putatively under selection across the 24 locations within the Maritimes. Furthermore, despite the overall low genetic diversity found in pairwise comparisons analysing oysters across estuarine gradients, all nine independent estuary‐specific analyses found significant SNP loci putatively under‐selection. In addition, a total of 12 SNP loci under selection in the analysis of estuarine gradient were found mutually between two estuaries (File S2).

Across all comparisons, structural annotation and subsequent GO characterization of these SNP loci resulted in the identification of functional physiological themes. The first major functional theme identified multiple markers mapping to cellular membrane function, ion transport and channel complexes. For example, in the analysis comparing all 24 locations in the Maritime provinces, SNP loci were annotated to transmembrane channel‐like protein 3, sodium‐coupled monocarboxylate transporter 1‐like, organic cation transporter protein‐like, and solute carrier family 22 member 4‐like. Similarly, in the analysis of estuarine gradients, SNP loci were annotated to solute carrier organic anion transporter family member (Richibucto); sodium leak channel non‐selective protein‐like (Bouctouche), Inositol 1,4,5‐trisphosphate receptor (Brudenell River), and a transporter (Caraquet). The identification of genes related to membrane function, ion transport and channel complexes may reflect selection on osmoconformation processes, a vital physiological requirement for oyster populations frequently exposed to estuarine salinity fluctuations (He et al. 2009; Ertl et al. 2019). Intriguingly, within the unique estuarine environment of the Bras d'Or Lakes, two SNP loci under selection were mapped to cholesterol dynamics (neutral cholesterol ester hydrolase 1‐like and Niemann‐Pick C1 protein‐like), which may be important for membrane fluidity. Cholesterol storage in membranes has been found to be important for other bivalves experiencing differing salinity regimes (Nemova et al. 2013; Ran et al. 2017).

The second functional theme in the analysis comparing all 24 locations in the Maritime provinces identified multiple SNP loci mapped to molecular chaperones and homeostatic regulators, including hypoxia up‐regulated protein 1, heat shock 70 kDa protein 12A‐like, and glutamate receptor 1. These genes have a role in molecular defence, regulating the unfolded protein response, and stabilizing cellular integrity during environmental stress (Kiang and Tsokos 1998). The third functional theme comprised a subset of SNP loci involved in cellular protein turnover pathways. These systems may also have a role in stress response to maintain quality control by tagging and degrading damaged or denatured proteins. In the analysis comparing all 24 locations in the Maritime provinces, SNP loci were annotated to E3 ubiquitin‐protein ligase rififylin‐like isoform X1, RING‐type and RBR‐type E3 ubiquitin transferases, metalloendopeptidases. The analyses across independent estuarine gradients also identified significant SNP loci in both Bouctouche and Grand River mapped to aminopeptidase. Interestingly, leucine aminopeptidase genes (LAP) have been found to be a marker of salinity gradients in bivalves (Koehn et al. 1976; Buroker 1983; Deaton et al. 1984; Nirchio and Pérez 1991). For example, Buroker (1983) found that *C. virginica* in the Gulf of Mexico from lower salinity environments contained LAP‐2^92^, compared to oysters in areas with higher salinity where LAP‐2^94^ was more common. It is therefore possible that the SNP loci annotated as aminopeptidase in the present study may represent a candidate marker associated with salinity‐related selection.

Finally, the fourth physiological theme involved several SNP loci identified to have a role in metabolic and energetic pathways, such as sphinganine‐1‐phosphate aldolase, maltase A3‐like, and N‐acetylneuraminate 9‐O‐acetyltransferase‐like for the analysis of all 24 locations in the Maritimes provinces, and beta‐N‐acetylhexosaminidase (Richibucto), phosphoenolpyruvate carboxykinase (Miramichi) for the analysis of estuarine gradients. Interestingly, previous studies have found physiological differences in metabolic and growth performance between different locations in shellfish in the Maritime provinces. For example, differences in metabolic and growth‐related performance have been reported in oysters from the Richibucto and Miramichi areas compared to other locations in New Brunswick. Méthé et al. (2020) studied metabolism, multi‐locus heterozygosity, and stress response of oysters from Miramichi, Richibucto, and Bouctouche and found that oysters were physiologically similar for most components; however, Richibucto oysters had a higher maximum metabolism (VO_2_max) compared to Bouctouche and Caraquet oysters. Mallet and Haley (1983) studied phenotypes of oyster larvae from crosses within and between locations in the Maritimes (Miscou, St. Simon, Miramichi, Bouctouche, Richibucto, and Shemogue) and reported significant differences in larval growth depending on the localities used. Specifically, they reported that oyster larvae originating from parental crosses using Miramichi oysters grew 35% slower compared to using Bouctouche oysters (Mallet and Haley 1983). Collectively, these studies suggest that oysters from Richibucto and Miramichi may exhibit distinct physiological characteristics relative to other Maritime populations, providing a potential biological context for the metabolic candidate loci identified in the present study.

Environmental conditions vary significantly between locations in the Maritime provinces in relation to temperature, salinity, currents, dissolved oxygen, and phytoplankton abundance, all of which can act as critical drivers of local genetic adaptation (Lande and Shannon 1996; Schmidt et al. 2008). The functional annotations found in the present study represent key insights into the genomic landscape of these populations, highlighting potential molecular mechanisms tied directly to local environmental adaptation. Examining these dynamics in the Gulf of St. Lawrence, Bernatchez et al. (2018) similarly demonstrated that environmental parameters (temperature, salinity, and turbidity) exerted pronounced impacts on both neutral and adaptive genetic diversity within native oyster beds. However, it is critical to note that the identification of interesting functional annotations among outlier SNP loci does not provide definitive empirical proof of contemporary environmental adaptation. It is critical to acknowledge that the candidate SNP loci isolated in this study were not explicitly assayed to determine if their presence results in structural or conformational alterations to the translated protein products. Furthermore, the downstream effects of genetic hitchhiking driven by linkage disequilibrium with adjacent non‐coding genomic regions remain unquantified (Barton 2000). Consequently, while these outlier markers provide interesting functional annotations, further validation is warranted to distinguish between causal adaptive mutations and neutral variation.

There are several evolutionary mechanisms that can be responsible for driving outlier SNP loci between regions, including genetic drift (Wright 1931), sweepstakes reproductive success (SRS; Hedgecock and Pudovkin 2011), and genetic bottlenecks created through severe losses in diversity (Luikart et al. 1998). These factors either represent biological features that commonly characterize the genomic landscapes of marine shellfish populations (i.e., genetic drift and sweepstakes dynamics), or may represent defining components of the documented regional history of the specific oyster beds evaluated in this study (i.e., loss of genetic diversity through mass mortality from disease epidemics such as Malpeque disease and extensive anthropogenic stock transfers). The present study examined some aspects of these factors. For example, isolation by distance analysis revealed a significant relationship between geographic and genetic distance (*p* = 0.0020, *R* ^2^ = 0.4462), suggesting that dispersal and gene flow, potentially influenced by genetic drift, contribute to the observed population differentiation. Although SRS was not explicitly tested, estimated effective population sizes (Ne = 973.3–3924.2) did not indicate evidence of severe contemporary demographic contraction, but these estimates alone cannot rule out episodic sweepstakes recruitment events. Observed heterozygosity estimates were comparable to those reported by Bernatchez et al. (2018) and did not provide strong evidence for recent population bottlenecks, which might otherwise be expected following severe mortality events or large‐scale stock transfers; however, there is evidence that historical stock transfers contributed to the contemporary genetic structure observed among some oyster populations. Taken together, the results suggest that the genomic landscape of Maritime oyster beds were shaped by a combination of local environmental pressures, demographic processes, historical anthropogenic interventions, and evolutionary forces. Consequently, while several candidate loci identified in this study may be associated with adaptive responses to environmental heterogeneity, additional functional validation will be required to distinguish adaptive variation from signatures generated by selection, demographic history, and neutral evolutionary processes.

### 4.4 Conclusions and Implications for Aquaculture and Ecological Management

This study, together with those of Bernatchez et al. (2018) and Vercaemer et al. (2010), provides insight on how large‐scale oyster translocations associated with the Malpeque disease epidemic have influenced population genetics over multiple generations. Using ddRAD‐seq, we characterized genetic diversity and population structure of wild eastern oysters from 24 locations across New Brunswick, Prince Edward Island, and Nova Scotia. Although overall genetic differentiation among Maritime populations was low (*F*_st_ = 0.012), analyses identified spatial genetic structure, including distinct ancestries associated with the Bras d'Or Lakes, Miramichi, and Richibucto populations. In contrast, several populations throughout the Gulf of St. Lawrence shared a common dominant ancestry, while coastal Cape Breton populations exhibited mixed ancestry. Within estuaries, genetic differentiation between upriver and downriver sites was minimal, suggesting ongoing genetic exchange, and does not support the hypothesis that ‘small relict’ populations exist at heads of river systems as suggested by Drinnan (1967). However, the identification of loci found putatively under selection within every estuary examined indicates that local adaptation may occur despite high levels of connectivity. Functional annotation of candidate loci highlighted biological processes associated with membrane transport, cellular stress response, protein turnover, and metabolism. This suggests that environmental factors such as salinity may contribute to adaptive divergence, but that a combination of regional history and historical anthropogenic interventions are also likely at play.

These results have important implications for contemporary oyster restoration and management within the Maritime provinces. The movement of oysters among regions has the potential to influence not only target restoration sites but also adjacent wild populations through larval dispersal and genetic exchange. Consequently, careful consideration should be given to the genetic composition and origin of broodstock used in restoration, aquaculture, and enhancement programs. This consideration is particularly important when evaluating the importation of non‐local oyster stocks, as translocations can affect population fitness in both positive and negative ways, especially near the edges of a species' range (Holtz et al. 2025). From a regulatory perspective, it is important to consider the genetic implications of stock importations, breeding programs, and restoration efforts, as evidenced from previous restoration efforts during the Malpeque disease epidemic, as these processes have the potential to alter wild genetic structure and diversity. A significant body of evidence has emerged on the implementation of mass oyster transfers for restoration and aquaculture on multiple oyster species, which includes successes and lessons learned (Beaumont et al. 2006; Thompson et al. 2017; Hornick and Plough 2019; Sutherland et al. 2020; Howie and Bishop 2021). For example, in the United States, hatchery‐based oysters have been transplanted in several areas (Hornick and Plough 2019; Turley et al. 2019); however, Hornick and Plough (2019) found that hatchery stocks alone resulted in reduced genetic diversity in offspring, whereas supplementing hatchery stocks with wild broodstock maintained genetic diversity in wild reefs. Studies on European flat oysters ( *Ostrea edulis* ) revealed significant genetic differentiation between regions despite mass transplantation efforts (Beaumont et al. 2006). In contrast, transplants of non‐native Pacific oysters (*Magellana gigas*) to British Columbia, Canada show minimal genetic differentiation across locations and countries (France, Japan). This difference may reflect the absence of wild oyster supplementation in Pacific oyster programs (Sutherland et al. 2020).

More than a century after the outbreaks of Malpeque disease on PEI (and more than 70 years in NB and NS), the emergence of MSX and Dermo in the Gulf of Saint Lawrence have once again resulted in significant impacts to wild oyster populations in the Maritimes. Significant efforts are ongoing in the region to obtain disease resistant oyster seed, including the establishment of local breeding programs and the transfer of MSX‐ and Dermo‐resistant oyster seed from the United States into Atlantic Canada (Atlantic Canada Opportunities Agency 2026); oyster reef restoration is also beginning to be explored (Clements et al. 2026). The US oyster locations were removed from our analysis of oyster populations in the Canadian Maritimes because the degree of genetic differentiation observed between US and Canadian oysters confounded downstream analysis (Figures S2, S3 in File S1). This genetic difference between US and Canadian populations, as well as the observed genetic clustering within the region, should be taken into consideration and further analysed prior to introduction of US reproductively viable non‐native stocks. Collectively, our findings emphasize the importance of integrating population genomics into oyster management and restoration planning. As disease pressures, restoration efforts, and aquaculture activities continue to expand across the Maritimes, maintaining genetic diversity and preserving locally adapted variation will be critical to supporting the long‐term resilience and sustainability of wild oyster populations.

## Funding

This work was supported by Fisheries and Oceans Canada, Program for Aquaculture Regulatory Research (PARR 2019‐G‐09).

## Ethics Statement

Ethical approval for the collection and use of oysters in this study was not required as bivalves are not considered under the purview of the Canadian Council of Animal Care.

## Conflicts of Interest

The authors declare no conflicts of interest.

## Supporting information


**Figure S1:** Genomic data missingness summary.
**Figure S2:** ADMIXTURE plots for various *k* values (*k* = 2–8) for all locations including the three USA locations.
**Figure S3:** Principal component analysis (PCA) illustrating patterns of genetic variation among individuals from all locations, including the three USA locations. The PCA was generated using a filtered SNP dataset consisting of 7740 SNP loci.
**Figure S4:** ADMIXTURE plots for various *k* values (*k* = 2–8) for all locations without the three USA locations.
**Table S1:** Analysis of molecular variance (AMOVA) among 24 locations of oysters ( *Crassostrea virginica* ), and 4 groups of oysters as identified by ADMIXTURE and PCA analysis.
**Table S2:** Linearized isolation by distance matrix.

**File S2:** Lists of candidate loci for each analysis including US samples.

**File S3:** Lists of candidate loci for each analysis excluding US samples.

**File S4:** Functional profiles derived from the descriptive review of gene ontology (GO) terms.

## Data Availability

Data underpinning the annotation of SNP loci under selection are provided in the Supporting Information. All ddRAD‐seq data will be submitted to the NCBI GenBank database upon publication. All other data and R code can be made available upon request.
