# Supplementary material for: Genetic Diversity and Structure Analysis of Wild Oysters (Crassostrea virginica) Within and Between River Systems Reveals Spatial Genetic Structure and Salinity‐Adaptive Variation Across the Canadian Maritimes
Source: Evol Appl. 2026 Oct 22;19(10):e70338. doi: 10.1111/eva.70338 (PMC13687925; doi:10.1111/eva.70338)
Supplement: Supplementary file 1 [file EVA-19-e70338-s001.docx]

**Supplementary Figures and Tables for:**

Genetic diversity and structure analysis of wild oysters (*Crassostrea virginica*) within and between river systems reveals spatial genetic structure and salinity-adaptive variation across the Canadian Maritimes

**Figure S1.** Genomic data missingness summary. Panel A shows the global distribution of missing data per SNP marker, and Panel B displays the average individual missing data percentage grouped by sample location.

**Figure S2.** ADMIXTURE plots for various *k* values (*k* = 2–8) for all locations including the three USA locations.

Figure S3. Principal component analysis (PCA) illustrating patterns of genetic variation among individuals from all locations, including the three USA locations. The PCA was generated using a filtered SNP dataset consisting of 7740 SNP loci.

**Figure S4.** ADMIXTURE plots for various *k* values (*k* = 2–8) for all locations without the three USA locations.

**Table S1.** Analysis of molecular variance (AMOVA) among 24 locations of oysters (*Crassostrea virginica*), and 4 groups of oysters as identified by ADMIXTURE and PCA analysis.

| Source of variation | Df | % of variation | P-value |
| --- | --- | --- | --- |
| **(A) All** | | | |
| Between group | 3 | 1.2 | 0.01 |
| Between location, within group | 23 | 0.14 | 0.01 |
| Between samples within location | 1442 | 23.4 | 0.01 |
| Within samples | 1469 | 75.1 | 0.01 |
| **(B) Neutral** | | | |
| Between group | 4 | 1.3 | 0.01 |
| Between location, within group | 19 | 0.18 | 0.01 |
| Between samples within location | 1361 | 25.1 | 0.01 |
| Within samples | 1385 | 73.3 | 0.01 |
| **(C) Under selection** | | | |
| Between group | 4 | 6.22 | 0.01 |
| Between location, within group | 19 | 0.923 | 0.01 |
| Between samples within location | 1361 | 27.2 | 0.01 |
| Within samples | 1385 | 65.5 | 0.01 |

**Table S2.** Linearized isolation by distance matrix. The upper portion of the matrix displays pairwise genetic distance values, while the lower portion of the matrix indicates the corresponding geographic distances. Colour shading highlights distance ranges between each pairwise comparison (Red shade = <30 km, Green shade = 30 – 250km, Blue shade = >250km)
